# Higher ventilatory responses during and after passive walking-like leg movement in older individuals

**DOI:** 10.1186/1880-6805-32-20

**Published:** 2013-11-08

**Authors:** Hisayoshi Ogata, Ikuyo Fujimaru, Keiko Yamada, Takaharu Kondo

**Affiliations:** 1 Department of Lifelong Sports for Health, College of Life and Health Sciences, Chubu University, 1200 Matsumoto-cho, Kasugai-shi, Aichi 487-8501, Japan

**Keywords:** Walking, Passive limb movement, Standing posture, Ventilation, Aging

## Abstract

**Background:**

Minute ventilation ($\overset{\cdot }{V}E$) during walking has been shown to be higher in older individuals than in young individuals, but the mechanisms underlying the higher ventilatory response is unclear. Central command and peripheral neural reflex are important neural control mechanisms underlying ventilatory response during exercise. Passive leg movement has been used to exclude the influence of central command due to the lack of voluntary activation of muscles. The aim of the present study was to compare the ventilatory response during and after passive walking-like leg movement (PWM) in young and older individuals.

**Methods:**

Eight young subjects (20 ± 2 years) and seven older subjects (70 ± 1 years) participated in this study. Subjects spent 7 minutes in a quiet standing (QS) position. Thereafter, they performed 14-minute rhythmic PWM at 1 Hz and this was followed by 7 minutes of QS.

**Results:**

$\overset{\cdot }{V}E$ values during pre-PWM QS were calculated as 1-minute averages using data obtained between 5 and 6 minutes. $\overset{\cdot }{V}E$ values at pre-PWM QS in the young and older groups were 8.4 ± 2.1 and 7.5 ± 1.2 l/minute, respectively. $\overset{\cdot }{V}E$ values increased significantly at the first minute of PWM to 11.4 ± 2.2 and 10.4 ± 2.5 l/minute in the young and older groups, respectively (*P* <0.001). In the young group, $\overset{\cdot }{V}E$ at the last minute of PWM (9.2 ± 2.0 l/minute) was not significantly different from that at pre-PWM QS due to a decline in $\overset{\cdot }{V}E$, whereas $\overset{\cdot }{V}E$ at the last minute of PWM in the older group (9.4 ± 2.2 l/minute) was still significantly higher (*P* <0.01). On the other hand, $\overset{\cdot }{V}E$ at the first minute of post-PWM QS (7.2 ± 1.8 l/minute) was significantly lower than that during pre-PWM QS in the young group (*P* <0.05) but not in the older group.

**Conclusions:**

Ventilatory response during and after PWM is higher in older individuals than in young individuals. This may be associated with a mechanism(s) other than central command. Our findings may explain part of the higher $\overset{\cdot }{V}E$ response while walking in older individuals.

## Background

Minute ventilation ($\overset{\cdot }{V}E$) during walking has been shown to be higher in older individuals than that in young individuals [1,2]. Since an increase in respiratory demand may compromise stability of the standing posture [3], it is important to clarify the mechanisms underlying higher $\overset{\cdot }{V}E$ response to walking in terms of gait stability and fall risk in older individuals.

Central command and peripheral neural reflex are important neural control mechanisms underlying ventilatory response during exercise [4]. To isolate the influence of peripheral neural reflex on ventilatory response, passive limb movement have been widely used because central command is minimized due to the lack of voluntary activation of muscles [5-7]. Bell and colleagues determined $\overset{\cdot }{V}E$ responses to 5-minute passive leg cycling movement and 5-minute passive leg extension movement in young individuals [5]. They found that $\overset{\cdot }{V}E$ increased abruptly after the onset of movement but gradually declined with time in both modes of movement. Although the mechanisms underlying this decline have not been elucidated, it is speculated that afferent feedback declines due to an adaptation of the receptors involved as the passive limb movement continues [5]. To date, there has been no study on time-dependent change in $\overset{\cdot }{V}E$ during passive walking-like leg movement (PWM) in young and older individuals. The decline of $\overset{\cdot }{V}E$ during PWM is possibly blunted in older individuals.

Post-exercise decrease in $\overset{\cdot }{V}E$ to its resting value is related to the removal of central command and afferent neural inputs from peripheral receptors [4]. Thus, it is assumed that $\overset{\cdot }{V}E$ response after passive movement is caused mainly by removal of afferent neural inputs. Bell and colleagues demonstrated a significant decrease in $\overset{\cdot }{V}E$ immediately after the end of passive leg extension movement despite the fact that $\overset{\cdot }{V}E$ level at the end of movement was almost the same as the pre-movement resting level [5]. These findings indicate that $\overset{\cdot }{V}E$ falls below the pre-movement level immediately after the end of movement, although Bell and colleagues did not report such a fall [5]. $\overset{\cdot }{V}E$ possibly falls below the pre-PWM level immediately after the end of PWM and such a fall may be different in older individuals.

The purpose of the present study was thus to compare the $\overset{\cdot }{V}E$ responses during and after PWM in young and older individuals.

## Methods

### Subjects

Eight healthy young subjects and seven healthy older subjects participated in this study. In recruiting subjects, an effort was made to match groups according to gender, height, weight and body mass index (Table 1). All of the subjects were nonsmokers. Participation was further restricted to generally robust individuals who had no major orthopedic, neurological, respiratory or cardiovascular disorders that might affect the results. This was done to avoid confounding effects of age-related pathologies according to previous studies [8-10]. Six of the seven subjects in the older group regularly performed exercise. Five subjects performed walking for more than 40 minutes a day and more than three times a week, and one subject performed yoga and stretching for about 1.5 hours more than once a week. In the young group, on the other hand, only two of the eight subjects regularly performed exercise.

**Table 1:** Characteristics of the subjects

	**Young group**	**Older group**
	**(*n*** **= 8; male = 4, female = 4)**	**(*n*** **= 7; male = 3, female = 4)**
Age (years)	20 ± 2	70 ± 1***
Height (m)	1.64 ± 0.10	1.58 ± 0.11
Weight (kg)	60.1 ± 8.0	52.9 ± 7.3
Body mass index (kg/m^2^)	22.3 ± 2.1	21.1 ± 1.9

***P <0.001 compared with values in the young group.

Voluntary consent for participation in this study was obtained from all subjects after they were informed of the purpose of the experiment, the procedure and possible risks. The study was conducted in accordance with the Helsinki Declaration and was approved by the Ethics Committee of Chubu University in Kasugai-shi, Aichi, Japan.

### Experimental protocols

The subjects refrained from eating for at least 3 hours before the test, from taking caffeine for at least 5 hours before the test, and from drinking alcohol and doing heavy exercise for 12 hours before the test. The temperature in the experimental room was set to 26°C.

Quiet standing (QS) and PWM were carried out using a commercially available device (Easy Stand Glider; Altimate Medical, Inc., Morton, MN, USA) as is described in detail elsewhere [11,12]. Briefly, this device enables subjects to change their posture from sitting to standing by pulling a built-in hydraulic lever. It took about 30 seconds to change from the sitting position to the standing position. The standing posture is stabilized by fixing the trunk, pelvis and knees using front, lateral and back trunk pads, lateral pelvic pads, and kneepads. Bilateral handles located in front of the trunk are linked to the footplates, thus allowing one leg to move forward while the other moves back by pushing and pulling the handles alternately. In the present study, an experimenter (HO) manually pulled the hydraulic lever and moved the handles.

First, subjects spent 7 minutes in a sitting position and this was followed by a 7-minute QS period to determine the baseline levels in sitting and standing states. Thereafter, they performed 14-minute rhythmic PWM at 1 Hz. The hip joint range of motion was set at 30°. After the PWM, the subjects spent 7 minutes in a QS position and this was followed by a 7-minute sitting period. The subjects were occasionally instructed to relax their body during the experiment.

During PWM, the experimenter always checked angle data displayed on an oscilloscope to maintain the predetermined pattern (that is, hip joint range of motion and swing frequency). The angle was measured by an electrogoniometer (MLTS700; AD Instruments Pty Ltd, Bella Vista, NSW, Australia) placed at the junction of the handle bar and the bar linked to a kneepad. The experimenter (HO) conducted a sufficient number of practice sessions before the main tests so that he could adjust the leg motion to the predetermined pattern by monitoring the angle data displayed on the oscilloscope.

### Measurements

Data on $\overset{\cdot }{V}E$, pulmonary oxygen uptake ($\overset{\cdot }{V}O_{2}$), carbon dioxide output ($\overset{\cdot }{V}CO_{2}$) and end-tidal carbon dioxide pressure (PETCO_2_) were obtained breath by breath using a respiratory gas analyzer (AE-300S; Minato Medical Science, Osaka, Japan). The averages of these data were calculated for each 15-second interval. Ventilatory parameters were measured by a hot-wire flow meter, and the flow meter was calibrated with a syringe of known volume (2.0 l). Oxygen and carbon dioxide concentrations were measured by a zirconium sensor and infrared absorption analyzer, respectively. The gas analyzer was calibrated by known standard gas (O_2_, 14.93%; CO_2_, 4.919%). Arterial carbon dioxide pressure was predicted from PETCO_2_ using the following equation [13], where PaCO_2pre_ is the predicted arterial carbon dioxide pressure:

$$\begin{array}{ll} \mathrm{PaC}O_{2\mathrm{pre}}\left(\mathrm{mmHg}\right))= & 5.5+0.90\times \mathrm{PETC}O_{2}\left(\mathrm{mmHg}\right)\\ & –0.0021\times \mathrm{tidal}\;\mathrm{volume}\left(\mathrm{ml}\right) \end{array}$$

An electrocardiogram was obtained at a sampling rate of 1,000 Hz using a bioamplifier (Dual BIO Amp; AD Instruments Pty Ltd) to determine the heart rate (HR) from R–R intervals.

Systolic and diastolic arterial blood pressures were determined noninvasively using an electro-sphygmomanometer (Tango+; Sun Tech Medical, Inc. Morrisville, NC, USA). A pneumatic cuff was fixed to the right upper arm, and Korotkov sound was detected by a lavalier microphone fixed on the left brachial artery. The sampling interval was set at 1 minute because it took about 30 seconds to terminate one data sampling, but the sampling interval was sometimes prolonged for several tens of seconds automatically for safety. Mean arterial blood pressure was calculated as the diastolic arterial blood pressure plus one-third of the pulse pressure.

For each of the cardiorespiratory variables except blood pressure, we calculated 1-minute averages at pre-PWM sitting (from 5 to 6 minutes), at pre-PWM QS (from 5 to 6 minutes), at the first minute of PWM, at the last minute of PWM, at the first, second, third, fifth and seventh minutes of post-PWM QS, and at the last 1 minute of post-PWM sitting. With regard to values of pre-PWM sitting and standing, since the experimenter told the subjects to change their posture and commence the leg movement during the last 1 minute of pre-PWM sitting and pre-PWM standing, respectively, it is possible that arousal or attention by this instruction affects ventilatory response [14]. Thus, we avoided using averages of the last 1 minute.

Data for cardiorespiratory variables were analyzed through a two-way repeated-measures analysis of variance with age category (young vs. older) and condition within an experimental trial (sitting vs. QS vs. PWM). If an interaction was found, Tukey’s honestly significant difference test was used to determine the difference in the values between two conditions within an experimental protocol. Differences in the values between the two groups were assessed using the unpaired *t* test. If a significant main effect for conditions without interaction was observed, data were analyzed using Tukey’s honestly significant difference test to identify the simple main effect for conditions when data obtained in the two groups at each time point were combined. If a significant main effect for age without interaction was observed, data were analyzed using the unpaired *t* test to identify the simple main effect for age when data obtained at all time periods in each age group were combined. Pearson’s correlation was used to express the strength of the relationship between magnitude of changes in $\overset{\cdot }{V}E$ during PWM and that in $\overset{\cdot }{V}O_{2}$ during PWM. *P* <0.05 was regarded as statistically significant. All data are presented as the mean ± standard deviation.

## Results

**Figure 1:**
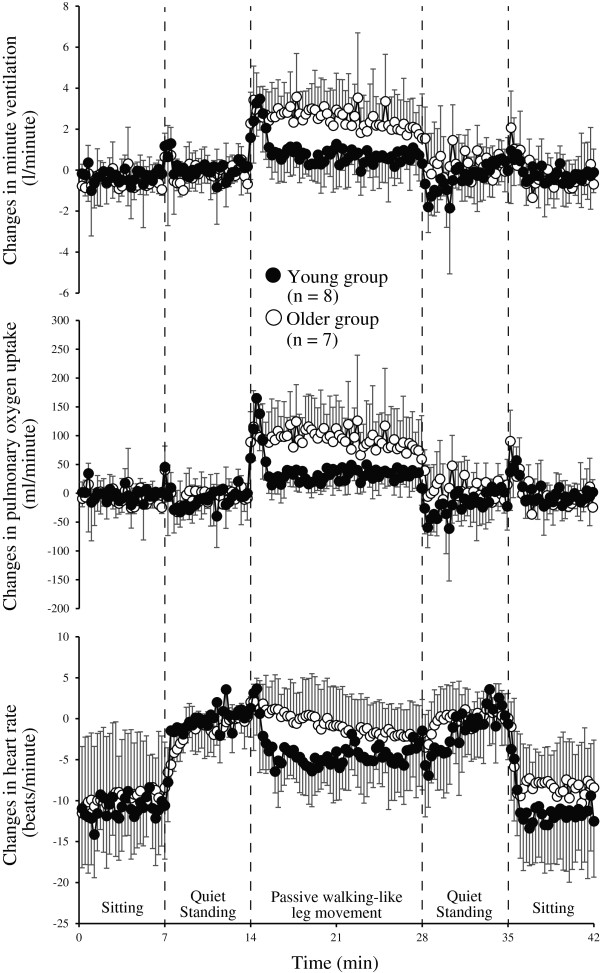
**Magnitudes of average change across subjects during sitting, quiet standing and passive walking-like leg movement in the young and older groups.** The 1-minute average during pre-PWM quiet standing (from 5 to 6 minutes) is set to zero.

Figure 1 shows average changes in $\overset{\cdot }{V}E$ and $\overset{\cdot }{V}O_{2}$ from the pre-PWM QS level (1-minute average using data from 5 to 6 minutes) across subjects using data averaged into 15-second epochs. General characteristics are as follows. First, although the initial $\overset{\cdot }{V}E$ responses after the onset of PWM were similar in the two groups, $\overset{\cdot }{V}E$ had declined to the pre-PWM standing level at the end of PWM in the young group, whereas $\overset{\cdot }{V}E$ remained elevated in the older group. Second, the levels of $\overset{\cdot }{V}E$ during QS immediately after the end of PWM were lower than those during pre-PWM QS in the young group but not in the older group. The pattern of change in $\overset{\cdot }{V}O_{2}$ was similar to that of $\overset{\cdot }{V}E$ in both groups.

Table 2 shows 1-minute average data for cardiorespiratory variables. In $\overset{\cdot }{V}E$, $\overset{\cdot }{V}O_{2}$ and $\overset{\cdot }{V}CO_{2}$, there was a significant main effect for conditions ($\overset{\cdot }{V}E$: *F*_(9,117)_ = 30.21, *P* <0.001; $\overset{\cdot }{V}O_{2}$: *F*_(9,117)_ = 41.88, *P* <0.001; $\overset{\cdot }{V}CO_{2}$: *F*_(9,117)_ = 35.78, *P* <0.001) and for interaction ($\overset{\cdot }{V}E$: condition × age, *F*_(9,117)_ = 2.90, *P* <0.01; $\overset{\cdot }{V}O_{2}$: condition × age, *F*_(9,117)_ = 3.66, *P* <0.05; $\overset{\cdot }{V}CO_{2}$: condition × age, *F*_(9,117)_ = 2.37, *P* <0.05). In HR, there were significant main effects for conditions (*F*_(9,117)_ = 24.39, *P* <0.001) and age (*F*_(1,13)_ = 6.15, *P* <0.05) without significant interaction. In PaCO_2pre_, there was only a significant main effect for conditions (*F*_(9,117)_ = 12.73, *P* <0.001).

**Table 2:** Cardiorespiratory variables during sitting, quiet standing and passive walking-like leg movement in the young and older groups

		**Sitting**		**Quiet standing**		**PWM**				**Quiet standing (recovery)**										**Sitting (recovery)**	
						**First 1 minute**		**Last 1 minute**		**First minute**		**Second minute**		**Third minute**		**Fifth minute**		**Seventh minute**			
$\overset{\cdot }{V}E$ (l/minute)																					
Young		8.0 ± 1.9		8.4 ± 2.1		11.4 ± 2.2	***,###	9.2 ± 2.0	*	7.2 ± 1.8	#	7.6 ± 1.7		7.9 ± 1.7		8.2 ± 1.5		8.8 ± 1.9		8.2 ± 1.5	
Older		6.9 ± 1.6		7.5 ± 1.2		10.4 ± 2.5	***,###	9.4 ± 2.2	***,##	8.0 ± 2.1		7.5 ± 1.7		8.0 ± 1.7		7.9 ± 1.2		7.9 ± 1.6		7.2 ± 1.8	
$\overset{\cdot }{V}O_{2}$ (ml/minute)																					
Young		204 ± 51		210 ± 54		337 ± 81	***,###	240 ± 63	*	169 ± 44	*,##	181 ± 42		190 ± 37		195 ± 41		209 ± 49		208 ± 45	
Older		160 ± 31		170 ± 23		272 ± 73	***,###	243 ± 70	***,###	179 ± 48		171 ± 41		182 ± 40		173 ± 18		176 ± 38		161 ± 23	†
$\overset{\cdot }{V}CO_{2}$ (ml/minute)																					
Young		169 ± 40		174 ± 49		278 ± 61	***,###	211 ± 51	***,##	148 ± 41		156 ± 40		159 ± 31		165 ± 32		179 ± 39		174 ± 34	
Older		137 ± 28		145 ± 20		241 ± 73	***,###	212 ± 66	***,##	163 ± 52		151 ± 40		157 ± 40		148 ± 18		151 ± 32		140 ± 23	†
PaCO_2pre_ (mmHg)																					
Young		38.6 ± 1.3	#	37.5 ± 1.4	*	38.9 ± 2.4		37.9 ± 2.0		36.4 ± 1.6	***	36.6 ± 1.7	***	36.8 ± 1.1	***	36.5 ± 1.3	***	36.1 ± 1.3	***,#	37.5 ± 1.9	*
Older		38.8 ± 2.7		37.5 ± 2.8		38.0 ± 2.4		37.8 ± 2.3		36.5 ± 1.7		36.7 ± 2.2		36.8 ± 2.0		36.4 ± 2.6		36.7 ± 2.7		37.6 ± 1.7	
HR (beats/minute)																					
Young		78 ± 9	###	88 ± 11	***	90 ± 10	***	86 ± 10	***	83 ± 10	***,#	84 ± 7	***	87 ± 7	***	88 ± 9	***	89 ± 10	***	77 ± 12	###
Older	†††	66 ± 8		75 ± 12		76 ± 13		73 ± 9		72 ± 10		75 ± 10		75 ± 10		75 ± 11		75 ± 12		66 ± 9	

A significant main effect for conditions, main effect for groups and their interaction were observed for minute ventilation ($\overset{\cdot }{V}E$), pulmonary oxygen uptake ($\overset{\cdot }{V}O_{2}$) and carbon dioxide output ($\overset{\cdot }{V}CO_{2}$). On the other hand, since a significant main effect for conditions without interaction was observed for PaCO_2pre_ and heart rate (HR), data were analyzed to identify the simple main effect for conditions when data obtained in the two groups at each time point were combined. In addition, since a significant main effect for age was observed for HR, data were analyzed to identify the simple main effect for age when data obtained at all time periods in each age group were combined. PaCO_2pre_, predicted arterial carbon dioxide pressure, calculated using data on end-tidal carbon dioxide pressure and tidal volume; PWM, passive walking-like leg movement.

*P <0.05, **P <0.01, and ***P <0.001, compared with the value at pre-PWM sitting.

^#^*P* <0.05, ^##^*P* <0.01, and ^###^*P* <0.001, compared with the value at pre-PWM standing.

^†^*P* <0.05 and ^†††^*P* <0.001, compared with the value in the young group.

In the young group, $\overset{\cdot }{V}E$ increased significantly at the first minute of PWM (*P* <0.001), while $\overset{\cdot }{V}E$ at the last minute of PWM was not significantly different from that at pre-PWM QS (Table 2). In the older group, on the other hand, $\overset{\cdot }{V}E$ increased significantly at the first minute of PWM (Table 2, *P* <0.001) as in the young group, but $\overset{\cdot }{V}E$ at the last minute of PWM was still significantly higher than that at pre-PWM QS (Table 2, *P* <0.01). The increase in $\overset{\cdot }{V}E$ at the last minute of PWM was accompanied by a significant increase in $\overset{\cdot }{V}O_{2}$ (Table 2, *P* <0.001). We determined the relationship between the magnitude of decline in $\overset{\cdot }{V}E$ from the first to last minutes of PWM and the magnitude of decline in $\overset{\cdot }{V}O_{2}$ from the first to last minutes of PWM across all subjects. The correlation coefficient obtained was 0.87 (*P* <0.001), indicating that the magnitude of decline in $\overset{\cdot }{V}E$ during PWM becomes lower with less decrease in $\overset{\cdot }{V}O_{2}$.

$\overset{\cdot }{V}E$ at the first minute of post-PWM QS was significantly lower than that at pre-PWM QS in the young group (Table 2, *P* <0.05). The magnitude of decrease was 1.2 ± 0.7 l/minute. $\overset{\cdot }{V}E$ from the second to seventh minutes of post-PWM QS was not significantly different from that at pre-PWM QS (Table 2). In the older group, on the other hand, $\overset{\cdot }{V}E$ during post-PWM QS was not significantly different from that at pre-PWM QS (Table 2). Despite this difference, the magnitudes of decrease in $\overset{\cdot }{V}E$ at the first minute of post-PWM QS from the level at the last minute of PWM in the young and older groups were 1.9 ± 0.8 and 1.4 ±1.6 l/minute, respectively. There was no significant difference between the values.

The HR, which had been increasing during QS, tended to decrease during PWM (Figure 1), although there was no significant difference in HR at pre-PWM QS and that at the last minute of PWM (Table 2). The HR tended to increase to the pre-PWM QS level after the end of PWM (Figure 1). However, the HR at the first minute of post-PWM QS was significantly lower than that at pre-PWM QS (Table 2, *P* <0.05).

PaCO_2pre_ at the first minute of post-PWM QS was not significantly different from that at pre-PWM QS (Table 2).

We compared values of mean arterial blood pressure recorded within 70 seconds after the end of PWM and within 5 to 6 minutes of pre-PWM QS in the young group. The former value was 88 ± 7 mmHg, while the latter value was 87 ± 9 mmHg. The paired *t* test showed no significant difference between the two values, whereas the paired *t* test showed a significant difference in $\overset{\cdot }{V}E$ recorded in the two time periods (*P* <0.01).

## Discussion

### Ventilatory responses during PWM

In the present study, $\overset{\cdot }{V}E$ increased rapidly after the onset of PWM in both the young and older groups. A similar change in $\overset{\cdot }{V}E$ was observed at the onset of passive limb movement in previous studies [5,6,15]. A rapid increase in $\overset{\cdot }{V}E$ is thought to be caused by peripheral neural reflex mechanisms arising from stimulation of group III and group IV afferents from moving limbs [15].

$\overset{\cdot }{V}E$, which had increased during the first minute of PWM, then decreased to the level at QS in the young group. A similar change in $\overset{\cdot }{V}E$ was found during 5-minute passive leg cycling movement in young individuals in a study by Bell and colleagues [5]. The mechanism underlying the decline of $\overset{\cdot }{V}E$ is thought to be an adaptation of afferent feedback from the moving limbs; as the passive limb movement continues, the afferent feedback declines due to an adaptation of the receptors involved [5].

On the other hand, although the initial increase in $\overset{\cdot }{V}E$ was similar to that observed in the young group, $\overset{\cdot }{V}E$ remained elevated above the level at QS in the older group. Group III and group IV mechanosensitive afferents innervate muscles and tendon tissues [16]. Since flexibility of the body, as evaluated by trunk flexion and extension tests, decreases with age [17], stiffness of muscle and tendon tissues could be higher in the older group. Increases in tissue stiffness may contribute to enhancement of the afferent feedback ventilatory response to passive leg movement if a given degree of limb deflection results in greater stimulation of mechanoreceptive afferents. However, if this is true, then the initial increase in $\overset{\cdot }{V}E$ may also be larger in the older group than in the young group. This was not the case in the present study.

The most plausible explanation is that enhanced oxygen consumption caused the elevated $\overset{\cdot }{V}E$ in the older group because elevated $\overset{\cdot }{V}E$ was accompanied by elevated $\overset{\cdot }{V}O_{2}$. The increased $\overset{\cdot }{V}O_{2}$ in the older group can be explained as follows. Bell and colleagues measured $\overset{\cdot }{V}O_{2}$ and electromyographic activity during passive leg cycling movement and passive leg extension movement, and found that simultaneous increases in $\overset{\cdot }{V}O_{2}$ and electromyographic activity occur only during passive leg cycling movement [5]. The increase in $\overset{\cdot }{V}O_{2}$ during PWM in the older group might thus have been due in part to involuntary muscle contraction of the legs. Obata and colleagues determined the effect of aging on stretch reflex responses of the soleus and tibialis anterior muscles at rest [18]. They found that stretch reflex amplitude of the soleus muscle did not differ between the older group and the young group, whereas the amplitude of long-latency stretch reflex of the tibialis anterior muscle, which is mediated partly by a transcortical reflex [19], was larger in the older group than in the young group. Therefore, the larger stretch reflex amplitude might have contributed to the increase in $\overset{\cdot }{V}O_{2}$ during PWM in the older group. In addition, involuntary contraction of the postural muscle might have been activated during PWM despite postural stabilization with the aid of a standing frame, resulting in an increase in $\overset{\cdot }{V}O_{2}$ in the older group.

### Heart rate response immediately after the end of PWM

The HR at the first minute of post-PWM QS was lower than that at pre-PWM QS. Cardiovascular responses to orthostatic stress can be simulated using lower body negative pressure (LBNP). Ishibashi and colleagues compared cardiovascular responses to sinusoidal LBNP (30-second and 180-second periods with average pressure of -25 mmHg) and -25 mmHg constant LBNP [20]. They demonstrated that an increase in HR was attenuated during a 30-second period of sinusoidal LBNP despite average pressure being the same, and they suggested that the attenuated increase in HR was due to the suppression of vagal responsiveness because the high-frequency component of HR variability was less reduced. These findings suggest that HR response to transient orthostatic stress can be attenuated due to attenuated vagal responsiveness. Since the high-frequency component of HR variability is increased during PWM compared with the level during QS [11], the high-frequency component of HR variability must have been decreased during QS after the end of PWM in the present study. However, if the decrease in the high-frequency component of HR variability (that is, the decrease in vagal activity) is transiently suppressed immediately after the end of PWM, an increase in HR may be suppressed.

### Possible mechanisms underlying decrease in ventilation immediately after the end of PWM

$\overset{\cdot }{V}E$ decreased transiently during QS immediately after the end of PWM in the young group. Ahn and colleagues demonstrated that 5-minute constant-load LBNP causes a decrease in $\overset{\cdot }{V}E$ with a reduction of cardiac output in humans [21]. Jones and colleagues found that ventilation changed linearly with right ventricular pressure, which is an index of right ventricular strain, when the strain was changed by increasing or decreasing cardiac output pharmacologically [22]. They thus suggested that changes in right ventricular strain act as a controller of ventilation via cardiac receptors and provide a link between ventilation and cardiac output. In the present study, HR at the first minute of post-PWM QS was significantly lower than that at pre-PWM QS. In addition, since PWM is thought to cause an increase in venous return due to the muscle pumping effect [11], cessation of PWM might have caused a sudden reduction of stroke volume. A reduction of cardiac output thus possibly causes a reduction of stimuli on cardiac receptors due to reduced right ventricular strain, resulting in a decrease in $\overset{\cdot }{V}E$ during QS immediately after the end of PWM. However, Ahn and colleagues also demonstrated that $\overset{\cdot }{V}E$ fell to the same degree at all levels of LBNP (-20 mmHg, –40 mmHg, –60 mmHg), although cardiac output was reduced proportionally to the level of the LBNP [21]. Thus, another mechanism possibly contributed to the decrease in $\overset{\cdot }{V}E$ in the present study.

A significant portion of group III and group IV afferent fibers lies in association with arterioles and venules in the muscle [16]. These fibers are stimulated by hemodynamic changes [16]. Fukuba and colleagues compared $\overset{\cdot }{V}E$ responses during recovery after exercise at intensities below and above the anaerobic threshold with and without femoral blood flow being occluded by a rapid cuff inflation [23]. They found a rapid decline in $\overset{\cdot }{V}E$ during the first 2 minutes of recovery from exercise at both exercise intensities in the occlusion condition. They thus suggested that mechanisms related to the hemodynamic effects of suddenly altered muscle perfusion contribute to the rapid fall of $\overset{\cdot }{V}E$. Since blood flow increases even during passive leg movement [24], the decrease in $\overset{\cdot }{V}E$ observed in the present study might have been related to suddenly decreased blood flow after the end of PWM.

Loading on arterial baroreceptors has been shown to decrease ventilation in animal and human studies [25,26]. One may expect that the transient increase in blood pressure after the end of PWM causes the decrease in $\overset{\cdot }{V}E$. Although we could not obtain a blood pressure value within 60 seconds after the end of PWM in any of the subjects due to technical limitation, data for mean arterial blood pressure obtained within 70 seconds after the end of PWM showed no significant difference from data obtained during pre-PWM QS. Thus, a contribution of the transient increase in blood pressure to the decrease in $\overset{\cdot }{V}E$ is unlikely.

Afroundeh and colleagues determined the relationship between $\overset{\cdot }{V}E$ and PaCO_2pre_ during recovery from light impulse-like exercise without metabolic acidosis and found that there was a significant positive relationship between $\overset{\cdot }{V}E$ and PaCO_2pre_ during the first 70 seconds of recovery [27]. From this finding, they suggested that arterial CO_2_ pressure drives $\overset{\cdot }{V}E$ via peripheral chemoreceptors during the first 70 seconds of recovery after light impulse-like exercise. On the other hand, the contribution of arterial CO_2_ pressure level to decrease in $\overset{\cdot }{V}E$ is unlikely in the present study because there was no significant difference between PaCO_2pre_ at pre-PWM QS and that at the first minute of post-PWM QS.

Although $\overset{\cdot }{V}E$ immediately after the end of PWM did not decrease below the level at pre-PWM QS in the older group, the magnitude of decrease in $\overset{\cdot }{V}E$ from the level at the end of PWM was not significantly different between the two groups. This suggests that the same mechanism(s) is operative for ventilator response in the two groups and that the elevated $\overset{\cdot }{V}E$ level during PWM, which may be caused by higher oxygen consumption, led to the absence of a decrease in $\overset{\cdot }{V}E$ below the level at pre-PWM QS in the older group.

### Implication

$\overset{\cdot }{V}E$ during walking has been shown to be higher in older individuals than that in young individuals [1,2]. Our findings suggest contribution of factors other than central command to higher $\overset{\cdot }{V}E$ response while walking. An increase in respiratory demand is thought to compromise stability of the standing posture [3]. On the other hand, Kita and colleagues found that $\overset{\cdot }{V}E$ increases during a postural task but that postural control practice reduces the increase in $\overset{\cdot }{V}E$ during the postural task [28]. If higher $\overset{\cdot }{V}E$ response during PWM in the older group is related to postural muscle activity, postural training may reduce the increase in $\overset{\cdot }{V}E$ during PWM, enabling further gait stability and reduced fall risks. Further study is needed to clarify this hypothesis.

## Conclusion

Ventilatory response during PWM is higher in older individuals than in young individuals due to higher oxygen consumption, which is possibly caused by involuntary muscle contraction. In the young individuals, $\overset{\cdot }{V}E$ immediately after the end of PWM fell below the pre-PWM level due to a neural factor(s), but the fall was suppressed in the older group due to elevated $\overset{\cdot }{V}E$ level during PWM. The findings of the present study may explain part of the higher ventilatory response while walking in older individuals.

## Abbreviations

HR: Heart rate; LBNP: Lower body negative pressure; PaCO2pre: Predicted arterial carbon dioxide pressure calculated from end-tidal carbon dioxide pressure and tidal volume; PETCO2: End-tidal carbon dioxide pressure; PWM: Passive walking-like leg movement in the standing posture; QS: Quiet standing; $\overset{\cdot }{V}CO_{2}$: Carbon dioxide output; $\overset{\cdot }{V}E$: Minute ventilation; $\overset{\cdot }{V}O_{2}$: Pulmonary oxygen uptake.

## Competing interests

The authors declare that they have no competing interests.

## Authors’ contributions

HO contributed to all of the works, including conception, design, acquisition of data, execution of the experiment (imposition of passive leg movement), analysis and interpretation of data, and writing the paper. IF and KY contributed to design and execution of the experiment (imposition of passive leg movement and management of risks of orthostatic intolerance). TK contributed to execution of the experiment (management of risks of orthostatic intolerance) and helped to draft the manuscript. All of the authors have read and approved the final manuscript.
